# Biochemical and economical effect of application biostimulants containing seaweed extracts and amino acids as an element of agroecological management of bean cultivation

**DOI:** 10.1038/s41598-020-74959-0

**Published:** 2020-10-20

**Authors:** Sławomir Kocira, Agnieszka Szparaga, Patryk Hara, Krzysztof Treder, Pavol Findura, Petr Bartoš, Martin Filip

**Affiliations:** 1 Department of Machinery Exploitation and Management of Production Processes, University of Life Sciences in Lublin, 20-950 Lublin, Poland; 2 Department of Agrobiotechnology, Koszalin University of Technology, 75-620 Koszalin, Poland; 3 Bonin Research Center, Plant Breeding and Acclimatization Institute-National Research Institute Laboratory of Molecular Diagnostic and Biochemistry, 76-009 Bonin, Poland; 4 Department of Machines and Production Biosystems, Slovak University of Agriculture in Nitra, 949 76 Nitra, Slovakia; 5 Department of Agricultural Machinery and Services, University of South Bohemia in České Budějovice, 370 05 České Budějovice, Czech Republic

**Keywords:** Agroecology, Plant breeding, Secondary metabolism, Plant sciences

## Abstract

The implementation of agronomic activities, based on the use of biostimulants, is an important element of agroecological practices. Therefore, comprehensive research was carried on the use of biostimulants. A field experiment was performed in 2016–2018 with common bean of Mexican Black cultivar. In particular growing seasons, bean plants were treated with Kelpak SL (seaweed extracts) and Terra Sorb Complex (free amino acids) in the form of single and double spraying with two solutions concentrations. According to the obtained data, application of biostimulants increased the yield of bean. Better results were observed after the use of Kelpak SL. The application of preparations influenced nutritional and nutraceutical quality of bean seeds. Terra Sorb Complex caused the highest increase in proteins level. In the light of achieved data, biostimulants in similar level decreased the starch accumulation. The most promising results, in the context of nutraceutical value of bean, were obtained in the case of increasing level of fiber. A positive impact of biostimulants on the seeds antioxidant potential was noted, expressed by the increased synthesis of phenolics, flavonoid, anthocyanins and antioxidant activities. Results of this study, directly indicate economic benefits from the use of biostimulants, which are extremely important to the farmers.

## Introduction

The limited pool of natural resources and damage caused to the natural environment by current agricultural practices have become the main drivers to discuss the principles of conventional agriculture. In this context, agroecology offers an important approach in agricultural systems design which takes account of interactions of their main biophysical, technical, and socioeconomic components^1,2^.

New technological tools have appeared in recent years that are dedicated to the sustainable development of agroecosystems^1^. It is obvious that crop production methods based solely on improving agricultural techniques and technologies (e.g. cultivation, fertilization, irrigation, etc.) are limited because they do not allow the biological potential of the grown crop to be fully exploited. In the face of the difficult task of preventing damage caused by abiotic and biotic factors in field crops, both the plant production and protection should be based, at the same time, on stimulating plant growth and development, while reducing risks posed to humans and the natural environment, as well as at providing safe high quality agricultural products (this means a strong reduction in the use of agrochemicals)^3–5^. To deal with these problems, conventional agriculture needs to increase its independence from chemical fertilizers and pesticides, which have a serious impact on the natural ecosystem and human health 3^6^. The use of biostimulants seems to be the best way to meet the urgent need for alternative organic methods based on new biologically active, environmentally friendly and safe substances^3,5,7–9^. Biostimulants are defined as products stimulating plants’ natural nutrition processes, as an effect of improving the plants’ nutrient use efficiency. Mechanism of action of biostimulants is connected also with increasing tolerance to abiotic stress, improving quality traits or increasing the availability of nutrients^10^. Their biological activity is due to the presence of, i.e. hormones, peptides, phenolic compounds, saccharides, and other organic components. In turn, commercial biostimulating preparations are mainly based on marine algae, protein hydrolysates, free amino acids or humic substances^11^.

Among the commercial biostimulants, worthy of special attention is Kelpak SL, produced from seaweeds representatives of the species *Ecklonia maxima* Osbeck, containing a variety of biologically active substances. Kelpak SL contains phytohormones: auxins and cytokinins, and also alginates, amino acids and small amounts of macro and microelements^12,13^. To the group of natural biostimulants it is also classified Terra Sorb Complex. This preparation is characterized by a high content of free amino acids (aliphatic amino acids, aromatic amino acids, acidic amino acids, and basic amino acids) synthesized via enzymatic hydrolysis^14^. Apart from the aforementioned compounds, Terra Sorb Complex contains also organic nitrogen, boron, magnesium, iron, zinc, manganese, molybdenum, and many microelements^14^.

The composition of these biostimulants may ensure appropriately high rate of plants growth and their increased resistance to stress factors, but it needs to be emphasized that each plant variety may respond differently to biostimulants application.

Over the past few years, plant growth biostimulants have successfully maintained on the agricultural inputs market as an agricultural practice that allows for the replacement or supplementation of mineral fertilizers^15–17^. Therefore, they represent a suitable alternative solution for the improvement of crop quality, while reducing environmental pollution^18–20^. The implementation of agronomic activities, based on the use of biostimulants, is an important element of agroecological practices, due to their huge potential in reducing the use of chemicals, saving energy, and providing farmers with new opportunities for both sustainable fertilization and disease control^1,15,21–23^. Treatment of plants with biostimulating preparations, containing active compounds may foster many unquestionable advantages. Besides promoting plant growth and development, their application also reduces costs and increases crop efficiency^24,25^. Effectiveness of biostimulants is determined by many factors, like i.e. appropriate choice of preparations, their dose, concentration and methods of application, but also species and cultivar of plants and environmental factors^26,27^. However, farmers rarely enthusiastically accept suggestions for the use of alternative crop management methods, especially at small-scale farms or in developing countries^28,29^. The main reason for reluctance to the implementation of biostimulating products or innovative crop protection strategies is no guarantee of success^30^. Skepticism of farmers regarding these alternative methods is also rooted in the belief that their effectiveness is low compared to the conventional chemicals^31,32^. Indeed, many studies have shown the biostimulants to exhibit variable effectiveness under real field conditions, as opposed to the promising and positive effects seen in controlled laboratory conditions^28^. Therefore, the benefits of biostimulant application must be clearly demonstrated, both in the form of research results and educational programs that will focus on real data obtained from field experiments^1,33^. According to many authors, this process can be complicated and lengthy, as it will include detailed knowledge of agronomic parameters and the design of adapted crop management techniques, with the right biostimulant product, applied at the right time and frequency, in combination with plant cultivars showing a positive response to its application^1,29,32^. In addition, this method of agroecological management of crops must meet farmers' requirements for ensuring optimal crop efficiency, while lowering input costs, and also for the compatibility of the biostimulants used, soil conditions, and last but not least, agricultural machinery and equipment^29^. Thus, the inclusion of biostimulants into agricultural practices largely depends on their economic importance compared to the conventional practices^1,29,32,33^.

According to Łączyński et al.^34^, a reduced yield of bean crop is noted in real field conditions due to, above all, its sensitivity to climate changes and stress factors. Therefore, it seems justified to use biostimulants containing active compounds that support bean growth and development. Although current predictions indicate a continuous increase in the use of biostimulants in both organic and sustainable agriculture, still little information can be found on the implementation of this agrotechnical treatment in bean cultivation. Our team has already conducted research on the influence of biostimulants on the yield and chemical composition of bean^14,19,35–37^ and soybeans seeds^7,9,38^. The results clearly showed that the plant response depended on the composition and doses of the preparations. However, depending on the tested plant cultivation, a different effect was also observed. This prompted us to choose a bean variety of dark seed coat color (Mexican black), due to the natural high content of nutraceutical ingredients.

In addition, an extremely important issue seems to be the assessment of the impact of biostimulant application methods not only on the level and quality of crop yield, but also on the economic efficiency of using such preparations. This article presents comprehensive research on the use of natural biostimulants containing seaweed extracts and free amino acids. This will allow verifying the hypothesis that their application can be a particularly valuable tool in agroecological and sustainable crop management.

## Results

**Figure 1:**
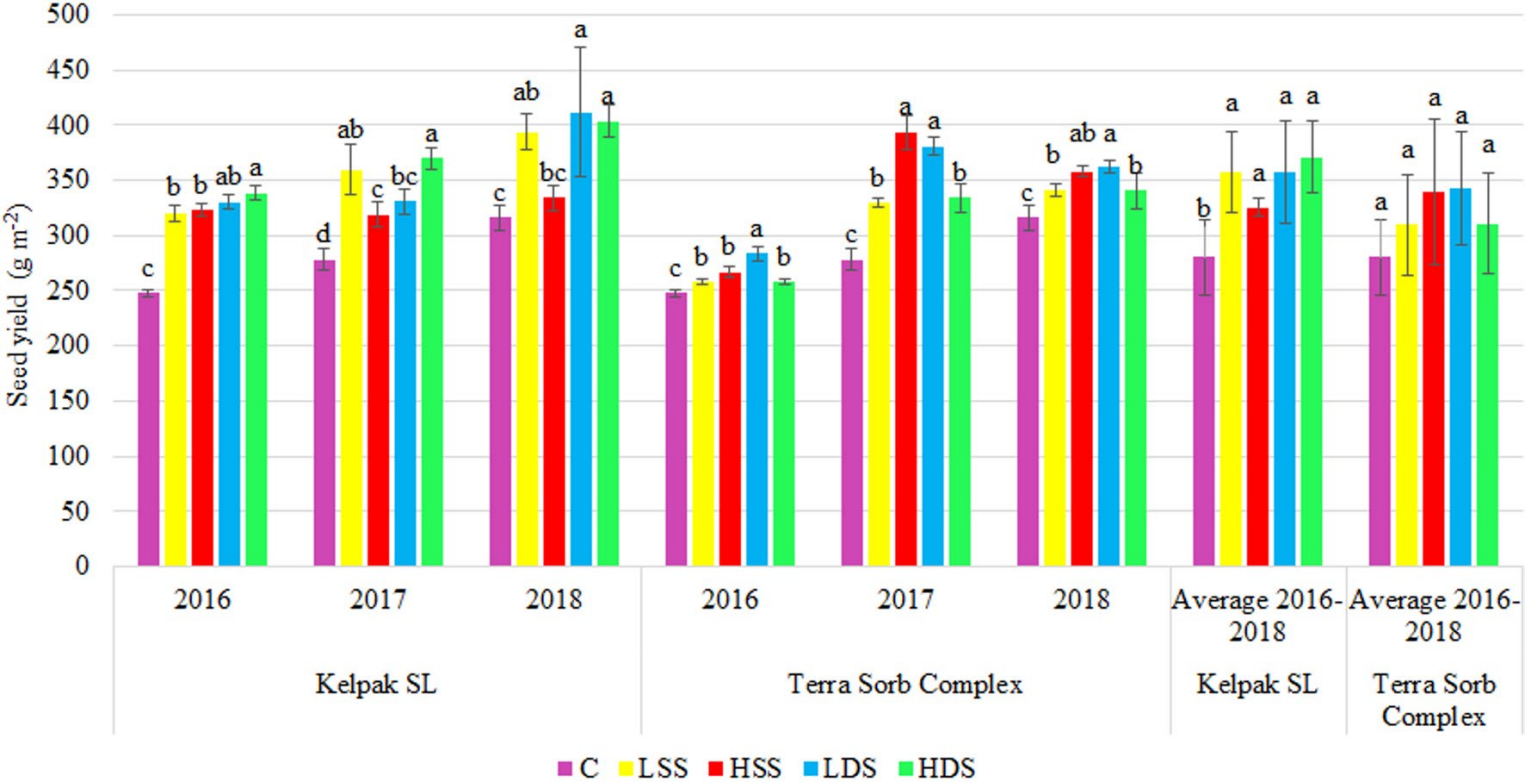
Effect of biostimulant treatment on the bean yield (g m^−2^). *C* control, *LSS* lower concentration single spraying, *LDS* lower concentration double spraying, *HSS* higher concentration single spraying, *HDS* higher concentration double spraying. Values followed by different small letters are significantly different at *p* < 0.05.

The results of a field experiment showed that the use of Kelpak SL and Terra Sorb Complex significantly increased bean yield in relation to the control treatment (C, Fig. 1). The most beneficial appeared to be double application of Kelpak SL in the higher concentration (HDS). In turn, analyzing the influence of the second tested product, the best effects were achieved upon double plant spraying with Terra Sorb Complex at its lower concentration (LDS).

The use of two biostimulants differing in composition contributed to a decreased proline content of bean seeds, regardless of the number of their applications and their concentrations (Table 1). A significant decrease in proline content compared to the control treatment (C) was demonstrated in the combination when plants were double sprayed with Kelpak SL in its lower concentration (LDS). The analysis of growing seasons demonstrated that proline concentration in 2017 was higher in two combinations compared to the control samples (C). The greatest increase in proline content in bean seeds was due to the double plant treatment with Kelpak SL in its higher concentration (HDS) and single spraying with the lower concentration solution (LSS) of this biostimulant.

**Table 1:** Effect of biostimulants treatment on nutraceutical quality.

Parameters	Biostimulant treatment	Biostimulant							
		Kelpak SL				Terra Sorb Complex			
		Season			Average ± SD	Season			Average ± SD
		2016	2017	2018		2016	2017	2018	
Protein (mg g^−1^ FM)	C	4970.7 ± 283.3b	5181.7 ± 604.9ab	4968.2 ± 224.2ab	5040.2 ± 122.6a	4970.7 ± 283.3a	5181.7 ± 604.9ab	4968.2 ± 224.2a	5040.2 ± 122.6b
	LSS	5484.1 ± 12.9c	4431.1 ± 265.8a	5246.5 ± 104.3b	5053.9 ± 552.3a	5803.6 ± 299.5b	5653.6 ± 375,9b	5712.3 ± 61.2b	5723.2 ± 75.6c
	HSS	4176.7 ± 252.8a	5883.9 ± 348.5b	4808.5 ± 238.2a	4956.4 ± 863.1a	4739.5 ± 285.1a	5238.8 ± 483.6ab	5022.0 ± 214.9a	5000.1 ± 250.4ab
	LDS	5337.5 ± 362.2bc	4669.4 ± 459.8a	5165.4 ± 170.1b	5057.4 ± 346.9a	5733.2 ± 577.9b	5384.1 ± 657.4ab	5460.4 ± 430.8b	5525.9 ± 183.5c
	HDS	5428.5 ± 258.6c	4453.3 ± 523.8a	5744.1 ± 80.8c	5208.6 ± 672.9a	4445.5 ± 331.9a	4685.8 ± 610.4a	4630.8 ± 214.1a	4587.4 ± 125.9a
FRAP (µM trolox/mL)	C	945.7 ± a117.0	1148.7 ± 93.3b	918.6 ± 83.7a	1004.3 ± 125.8a	945.7 ± 117.0a	1148.7 ± 93.3a	918.6 ± 83.7a	1004.3 ± 125.8a
	LSS	1023.7 ± 186.1a	1199.8 ± 38.2b	1098.3 ± 74.4c	1107.3 ± 55.4a	973.0 ± 133.8a	1068.7 ± 83.4a	1020.4 ± 42.3b	1020.7 ± 47.8a
	HSS	834.9 ± 49.8a	1179.6 ± 57.4b	941.7 ± 54.9ab	985.4 ± 176.4a	1174.9 ± 37.3b	1077.4 ± 141.0a	1122.9 ± 38.8c	1125.1 ± 48.8a
	LDS	1308.8 ± 73.8b	1083.0 ± 122.0ab	1061.8 ± 95.1bc	1151.2 ± 136.9a	852.4 ± 90.5a	1350.2 ± 135.4b	1108.0 ± 57.0bc	1103.5 ± 248.9a
	HDS	950.1 ± 150.8a	1000.2 ± 58.8a	1098.1 ± 61.2c	1016.1 ± 75.3a	1186.0 ± 109.6b	1121.5 ± 59.3a	1160.8 ± 54.1c	1156.1 ± 32.5a
ABTS (µM trolox/mL)	C	946.1 ± 89.2a	1003.1 ± 426.8a	955.4 ± 74.6a	968.2 ± 30.6a	946.1 ± 89.2a	1003.1 ± 426.8a	955.4 ± 74.6a	968.2 ± 30,6a
	LSS	1038.2 ± 160.5a	1349.4 ± 90.7a	1193.0 ± 83.4b	1193.5 ± 155.6a	1217.7 ± 216.5c	1169.1 ± 197.0a	1187.9 ± 45.0b	1191.6 ± 24.5a
	HSS	1089.1 ± 157.4a	1406.5 ± 114.0a	1134.2 ± 120.1ab	1209.9 ± 171.7a	1186.1 ± 110.2bc	1168.3 ± 709.8a	1173.8 ± 110.8b	1176.1 ± 9.1a
	LDS	1418.2 ± 204.8b	1210.9 ± 494.9a	1249.4 ± 229.9b	1292.8 ± 110.3a	889.3 ± 73.0a	1310.1 ± 276.4a	1089.8 ± 106.2ab	1096.4 ± 210.5a
	HDS	1158.7 ± 180.9ab	1143.0 ± 113.4a	1303.6 ± 89.6b	1201.8 ± 88.6a	968.3 ± 100.7ab	1247.1 ± 338.8a	1119.4 ± 33.7b	1111.6 ± 139.5a
Proline (µM mL^−1^)	C	5.912 ± 0.119b	4.982 ± 0.335ab	5.465 ± 0.272b	5.453 ± 0.465b	5.912 ± 0.119b	4.982 ± 0.335a	5.465 ± 0.272b	5.453 ± 0.465a
	LSS	4.730 ± 0.198a	5.132 ± 0.298ab	4.866 ± 0.282a	4.909 ± 0.204ab	5.185 ± 0.544a	4.502 ± 0.274a	4.917 ± 0.298a	4.868 ± 0.344a
	HSS	4.754 ± 0.204a	4.735 ± 0.539ab	4.916 ± 0.290a	4.801 ± 0.100ab	5.025 ± 0.306a	4.708 ± 0.666a	4.890 ± 0.254a	4.874 ± 0.159a
	LDS	4.775 ± 0.986a	4.512 ± 0.122.0a	4.682 ± 0.223a	4.656 ± 0.133a	4.821 ± 0.536a	4.938 ± 0.648a	4.830 ± 0.297a	4.863 ± 0.065a
	HDS	5.256 ± 0.303ab	5.230 ± 58.8b	4.870 ± 0.363a	5.119 ± 0.216ab	5.148 ± 0.359a	4.918 ± 0.622a	4.986 ± 0.167a	5.017 ± 0.118a

*C* control, *LSS* lower concentration single spraying, *LDS* lower concentration double spraying, *HSS* higher concentration single spraying, *HDS* higher concentration double spraying. Means in the columns, concerning the selected traits, followed by different small letters are significantly different at *p* < 0.05.

Plant treatment with Terra Sorb Complex biostimulant also resulted in a decreased proline content of bean seeds compared to the control treatment (C). Insignificant differences were noted in the extent of this amino acid reduction in the second growing season. On the other hand the highest decrease in proline content was determined in 2016 and 2018 after double foliar application of Terra Sorb Complex, in the lower concentration of working solution (LDS).

The application of Terra Sorb Complex in the lower concentrations contributed to an increase in protein concentration of bean seeds compared to the control treatment (C). Its working solutions used in the higher concentrations decreased protein content of the seeds of the double treated plants (HDS) in each year of the experiment (Table 1).

In contrast, analyzing the average results (2016–2018), there were no significant changes in protein content of the seeds in the case of plants treated with Kelpak SL. Increased protein content compared to the control treatment (C) in 2016 and 2018 was demonstrated in seeds from two combinations studied, i.e. after single spraying with biostimulant in lower concentration (LSS) and double spraying at higher concentration (HDS). In addition, protein content of bean seeds noted in the growing season of 2016 was higher compared to the other study years. An opposite observation was made after the use of Kelpak SL in 2017, where the content of protein increased only after single foliar application of 1.0% Kelpak SL solution (LDS). In the other tested combinations, plant treatment with Kelpak SL contributed to a decrease in protein content in the seeds compared do the control samples (C).

The antioxidative activity of bean was determined based on the FRAP test, the ability to scavenge ABTS + radical, and reducing power. Considering been seeds capability to reduce Fe (III) to Fe (II) (FRAP value), plant treatment with Terra Sorb Complex biostimulant contributed to an increased value of this parameter compared to the control (Table 1). An exception was found for the single plant spraying (LSS, 2017) and double plant treatment (LDS, 2016) with this preparation in its lower concentration. A significant increase in the FRAP value was noted after both single and double application (LDS and HDS) of Terra Sorb Complex biostimulant in the higher concentration (in 2016 and 2018). In contrast, meteorological conditions of the growing season of 2017 had a different effect on the FRAP. The significant increase in the FRAP value (by 14.20% compared to the control) was determined after double foliar application of Terra Sorb Complex in the lower concentration (LDS).

In contrast, after the application of the Kelpak SL biostimulant, high variability in the FRAP value was observed. Plant treatment with Kelpak SL biostimulant in 2016 year in the form of a double spraying in its lower concentration (LDS) contributed to FRAP significant increasing compared to the seeds from other combinations. In the 2017 FRAP decreased after double plant treatment with both concentrations of this biostimulant (LDS and HDS). The most interesting results were noted in 2018. The significantly higher FRAP values were observed in all tested combinations of plant treatments with Kelpak SL, compared to the control (C).

Considering the effect of the biostimulants tested on the capability of bean seed extracts to scavenge the ABTS cation radical, an increase was demonstrated in the value of this parameter regardless of preparation used, the number of its applications, and its concentration, compared to the control treatment (C). The greatest increase in the capability to inactivate ABTS+ was observed for the seeds from plants double sprayed with Kelpak SL in its higher (HDS, 2018) and lower concentration (LDS, 2016) (Table 1). In the second year of the field experiment, capability of seed extracts to scavenge the ABTS cation radical was higher for the samples from plants treated with Kelpak SL but the statistical analysis showed that the obtained results did not differ from each other. The application of Terra Sorb Complex biostimulant resulted in similar values of this trait in almost all combinations, except for the seeds from plants double sprayed with its solution in the lower concentration (LDS, 2016). In this combination, the increase in the antioxidative activity measured in the ABTS system were the smallest. Likewise in the FRAP test, meteorological conditions occurring in the growing seasons contributed to the changes in antiradical properties of bean seeds. As in the case of the Kelpak SL, Terra Sorb Complex application in 2017, caused an increase in the ABTS value in all tested combinations compared to the control (C). However, there were no statistically significant differences between the observed results.

Each of the biostimulants used contributed to an increase in the total polyphenols content (TPC) in bean seeds compared to the control treatment (Table 2). The greatest, over twofold increase in TPC was determined after single plant spraying with Kelpak SL in its lower concentration (LSS). In 2017, this combination caused the highest TPC compared to the other combinations. In turn, the smallest increase in TPC, compared to the non-treated plants (C), was determined after double application of Terra Sorb Complex in its lower concentration (LDS). The other treatments of plants with this preparation caused only small differences in the polyphenols content between the seeds. In addition, the total contents of polyphenolic compounds varied in particular growing seasons depending on the meteorological conditions. Values of TPC determined in 2017 were generally higher than these assayed in the other study years.

**Table 2:** Effect of biostimulants treatment on the antioxidant potential in common bean seeds.

Parameters	Biostimulant treatment	Biostimulant							
		Kelpak SL				Terra Sorb Complex			
		Season			Average ± SD	Season			Average ± SD
		2016	2017	2018		2016	2017	2018	
Anthocyanins (mg g^−1^ DM)	C	0.010 ± 0.001a	0.007 ± 0.01a	0.011 ± 0.001a	0.009 ± 0.002a	0.010 ± 0.001a	0.007 ± 0.001a	0.011 ± 0.001a	0.009 ± 0.002a
	LSS	0.393 ± 0.012c	0.516 ± 0.015d	0.306 ± 0.009d	0.405 ± 0.105b	0.226 ± 0.010c	0.564 ± 0.015d	0.382 ± 0.033c ± d	0.391 ± 0.169b
	HSS	0.218 ± 0.011b	0.378 ± 0.013b	0.201 ± 0.005b	0.266 ± 0.098b	0.418 ± 0.014d	0.320 ± 0.014b	0.348 ± 0.053c	0.362 ± 0.051b
	LDS	0.208 ± 0.013b	0.402 ± 0.010c	0.208 ± 0.007b	0.273 ± 0.112b	0.176 ± 0.012b	0.309 ± 0.012b	0.260 ± 0.040b	0.249 ± 0.067b
	HDS	0.441 ± 0.010d	0.372 ± 0.007b	0.274 ± 0.007c	0.362 ± 0.362b	0.534 ± 0.010e	0.354 ± 0.008c	0.438 ± 0.018d	0.442 ± 0.090b
Total flavonoids (mg g^−1^ DM)	C	1.166 ± 0.003a	1.095 ± 0.005a	1.210 ± 0.011a	1.157 ± 0.58a	1.166 ± 0.003a	1.095 ± 0.005a	1.210 ± 0.011a	1.157 ± 0.058a
	LSS	4.739 ± 0.089b	5.048 ± 0.078e	3.536 ± 0.050c	4.441 ± 0.798a	1.992 ± 0.081b	7.354 ± 0.119d	4.660 ± 0.062b	4.669 ± 2.681ab
	HSS	4.767 ± 0.106b	1.378 ± 0.125b	3.395 ± 0.076b	3.180 ± 1.705a	5.900 ± 0.110d	4.702 ± 0.146c	4.986 ± 0.770b	5.196 ± 0.626b
	LDS	6.216 ± 0.050c	4.219 ± 0.069d	3.695 ± 0.042d	4.710 ± 1.330a	4.686 ± 0.094c	4.384 ± 0.074b	4.669 ± 0.323b	4.580 ± 0.169ab
	HDS	10.262 ± 0.079d	3.914 ± 0.139c	4.323 ± 0.064e	6.166 ± 3.553a	7.386 ± 0.121e	4.850 ± 0.098c	6.051 ± 0.178c	6.096 ± 1.269b
Reducing power (mg TE g^−1^ DM)	C	2.459 ± 0.009b	2.967 ± 0.007a	2.638 ± 0.051	2.688 ± 0.258a	2.459 ± 0.009c	2.967 ± 0.007b	2.638 ± 0.051b	2.688 ± 0.258a
	LSS	2.306 ± 0.123b	2.932 ± 0.125a	2.882 ± 0.072c	2.707 ± 0.348a	2.318 ± 0.107bc	0.718 ± 0.093a	1.674 ± 0.380a	1.570 ± 0.805a
	HSS	3.060 ± 0.131c	3.842 ± 0.054c	3.299 ± 0.093d	3.400 ± 0.401a	2.727 ± 0.109d	4.740 ± 0.122c	3.640 ± 0.255c	3.702 ± 1.008a
	LDS	3.367 ± 0.062d	4.843 ± 0.133d	3.380 ± 0.055d	3.863 ± 0.849a	1.967 ± 0.116a	4.776 ± 0.117c	3.475 ± 0.237c	3.406 ± 1.406a
	HDS	1.662 ± 0.129a	3.386 ± 0.130b	2.501 ± 0.064a	2.516 ± 0.862a	2.224 ± 0.085b	7.008 ± 0.121d	4.541 ± 0.198d	4.591 ± 2.392a
Total phenols (mg g^−1^ DM)	C	23.555 ± 0.027a	26.252 ± 0.035a	25.083 ± 0.353a	24.963 ± 1.353a	23.555 ± 0.027a	26.252 ± 0.035a	25.083 ± 0.353a	24.963 ± 1.353a
	LSS	36.261 ± 0.577d	44.145 ± 0.683e	36.834 ± 0.435d	39.080 ± 4.369c	26.912 ± 0.403b	38.853 ± 0.406e	32.541 ± 0.799c	32.769 ± 5.974a
	HSS	29.849 ± 0.270b	31.656 ± 0.453c	31.496 ± 0.265c	31.000 ± 1.00ab	37.730 ± 0.669d	29.109 ± 0.362b	32.853 ± 1.339c	33.230 ± 4.323a
	LDS	32.770 ± 0.492bc	29.112 ± 0.697b	29.820 ± 0.563b	30.567 ± 1.940ab	24.090 ± 0.510a	33.207 ± 0.553c	29.069 ± 1.129b	28.789 ± 4.565a
	HDS	35.385 ± 0.277c	34.455 ± 0.473d	31.450 ± 0.106c	33.763 ± 2.056bc	31.894 ± 0.506c	35.844 ± 0.387d	33.831 ± 0.171c	33.856 ± 1.975a

*C* control, *LSS* lower concentration single spraying, *LDS* lower concentration double spraying, *HSS* higher concentration single spraying, *HDS* higher concentration double spraying. Means in the columns, concerning the selected traits, followed by different small letters are significantly different at p < 0.05.

The content of flavonoids in bean seeds was determined by both the number of treatments and concentrations of biostimulants (Table 2). In the case of Terra Sorb Complex, a significant increase in their content was determined upon each kind of treatment. Double plant treatment with this preparation in its higher concentration (HDS) caused the highest increase of this bioactive compounds in seeds (in 2016 and 2018). The similar results were observed in 2017, but as an effect of single plant spraying with Terra Sorb Complex in lower concentration (LSS).

Plant treatment with the preparation containing extracts from *Ecklonia maxima* caused an increase in flavonoid content of the seeds compared to the control treatment (C). However, it needs to be emphasized that after Kelpak SL application, similar dependencies, in the context of flavonoids concentration, were observed as those noted after the plant treatments with Terra Sorb Complex.

The analysis of anthocyanins demonstrated that their concentration in bean seeds depended not only on biostimulant type, number of its applications, and its concentration, but also on meteorological conditions occurring in individual growing seasons (Table 2). The greatest increase in the content of these compounds was determined in 2017 after single plant spraying with Kelpak SL and Terra Sorb Complex in their its lower concentration (LSS). The different effect was observed in the first year of the field experiment. The double application of these biostimulants in their higher concentrations (HDS) increased anthocyanins content of the seeds compared to the control treatment (C). In the case of the last year of the experiment the highest increase in this bioactive compounds was noted after single plant treatment with Kelpak SL in its lower concentration (LSS) and after double plant spraying with Terra Sorb Complex in higher concentration (HDS).

The double application of Terra Sorb Complex biostimulant in the higher concentration (HDS) caused increase in the reducing power of bean seeds compared to the control samples (C). A significant decrease in the value of this trait was determined for the seeds from plants single sprayed with this preparation in the lower concentration (LSS, Table 2). In 2016 the value of reducing power increased after single plant spraying with Terra Sorb Complex (HSS), while the similar tendency was observed in 2017–2018, but after double plant treatment with this biostimulant in higher tested concentration (HDS).

The Kelpak SL biostimulant had an opposite effect on the reducing power of bean seeds, when compared the same combinations from the experiment with Terra Sorb Complex. The greatest, increase in the value of this parameter compared to the control samples was noted after its double application in the lower concentration (LDS).

A complex evaluation of the effect of biostimulants tested on the antioxidative properties of bean seeds enabled concluding that Terra Sorb Complex was more effective compared to Kelpak SL. In addition, the meteorological conditions occurring in the growing season of 2017 contributed to the highest values noted in the antiradical tests.

**Figure 2:**
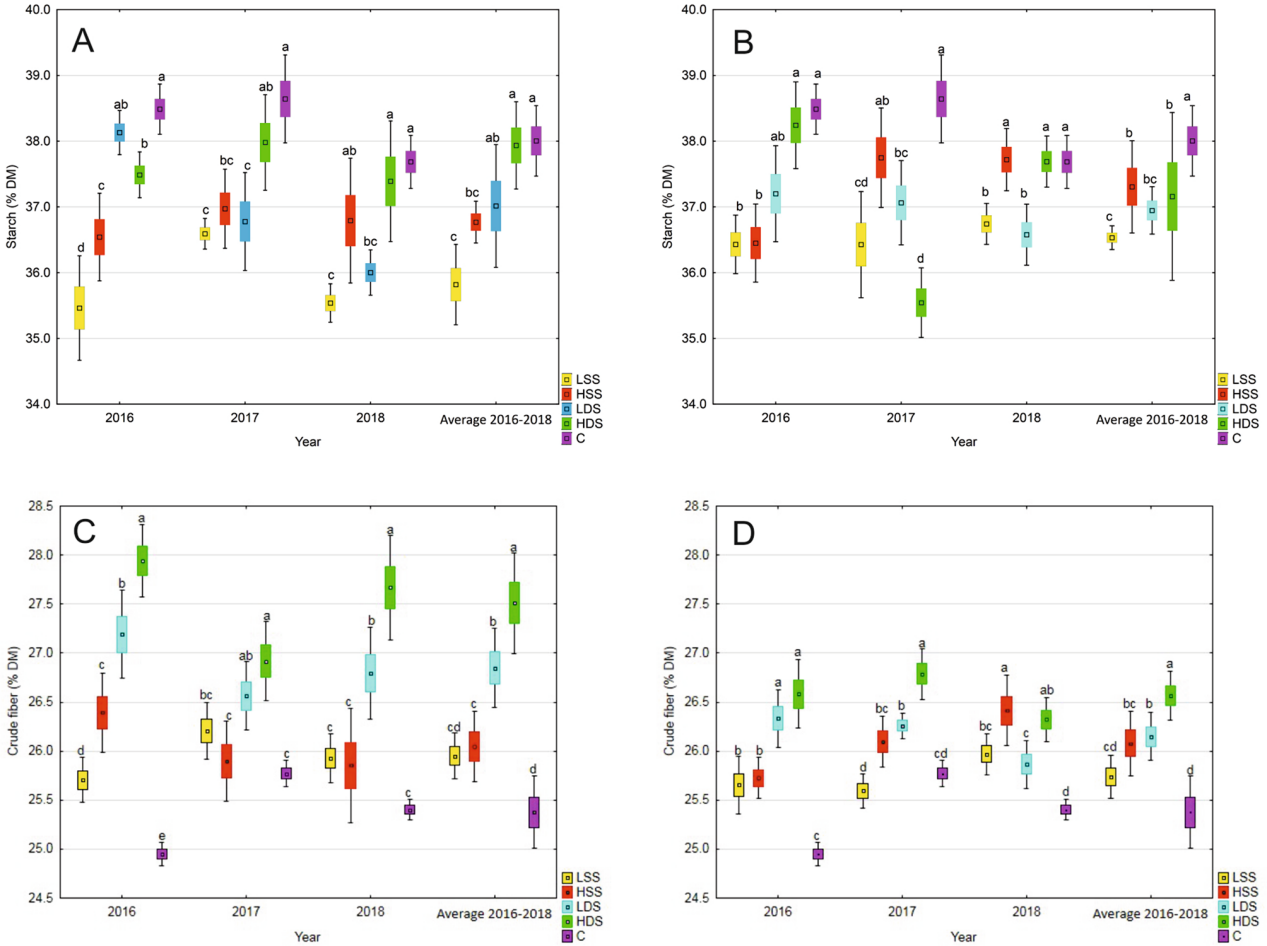
Effect of biostimulant treatment on the starch [(**A**) Kelpak SL treatment, (**B**) Terra Sorb Complex treatment] and crude fiber accumulation [(**C**) Kelpak SL treatment, (**D**) Terra Sorb Complex treatment] in bean seeds. *C* control, *LSS* lower concentration single spraying, *LDS* lower concentration double spraying, *HSS* higher concentration single spraying, *HDS* higher concentration double spraying. Means in the figures, concerning the selected traits, followed by different small letters are significantly different at *p* < 0.05. Figure created by using Statistica software 13.3 (TIBCO Software Inc., USA).

According to the obtained data (Fig. 2A) the application of Kelpak SL biostimulant did not induce the synthesis of starch in seeds. The level of this trait decreased in seeds from plant treated with tested biostimulant. The lowest content of starch was noted as an effect of single foliar application of Kelpak SL in its lower concentration (LSS). The similar to the control samples (C), but statistically different results were observed after double plant treatment with this preparation in its higher (HDS, 2017–2018) and lower (LDS, 2016) concentration.

In the case of Terra Sorb Complex (Fig. 2B), starch content in bean seeds generally was the highest in control samples (C). In 2016 the level of this trait determined after double application of this biostimulant in its higher concentrations (HDS), was similar to value in seeds from non-treated plants (C). Whereas in 2017, bean plants respond to this kind of Terra Sorb Complex treatment (HDS) with a highest decreased in starch accumulation. An opposite observation was made in 2018, where the content of starch did not statistically differ between control (C) and seeds from plants treated with biostimulant in its higher concentration.

Application of Kelpak SL generally contributed to an increased content of crude fiber in bean seeds compared to the control treatment (Fig. 2C). The highest values was obtained in 2016, when the foliar application of tested biostimulant in common bean cultivation caused significantly changes in crude fiber, especially after double plant spraying with solutions in both concentration (LDS and HDS). The presented data showed also that single plant treatment with Kelpak SL in higher concentration (LDS) did not induce any changes in fiber content in seeds compared to the control samples (C).

The similar observations were made for Terra Sorb Complex biostimulant which caused an increase in fiber content in seeds from all combinations compared to the control seeds (Fig. 2D). The greatest increase in fiber content in bean seeds was due to the double plant treatment with Terra Sorb Complex in its higher concentration (HDS, 2016–2017) and single spraying with the same solution (LDS, 2018). The obtained data also showed the increase in the crude fiber content in bean seeds in the first and second year of the experiment, which represented plant response to the double application of this biostimulant in the lower concentration (LDS).

**Figure 3:**
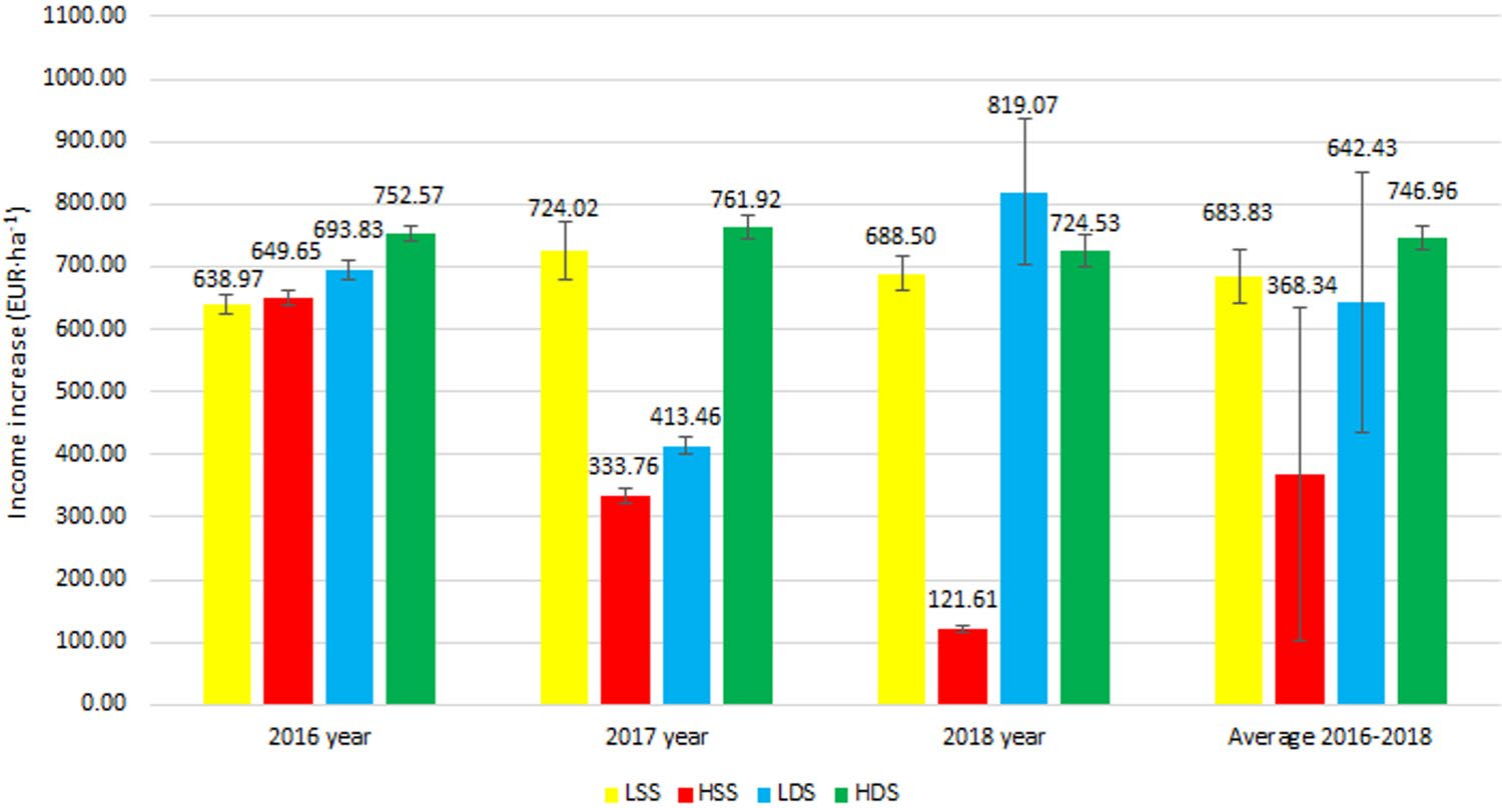
Economic effects of biostimulant Kelpak SL use. *C* control, *LSS* lower concentration single spraying, *LDS* lower concentration double spraying, *HSS* higher concentration single spraying, *HDS* higher concentration double spraying.

**Figure 4:**
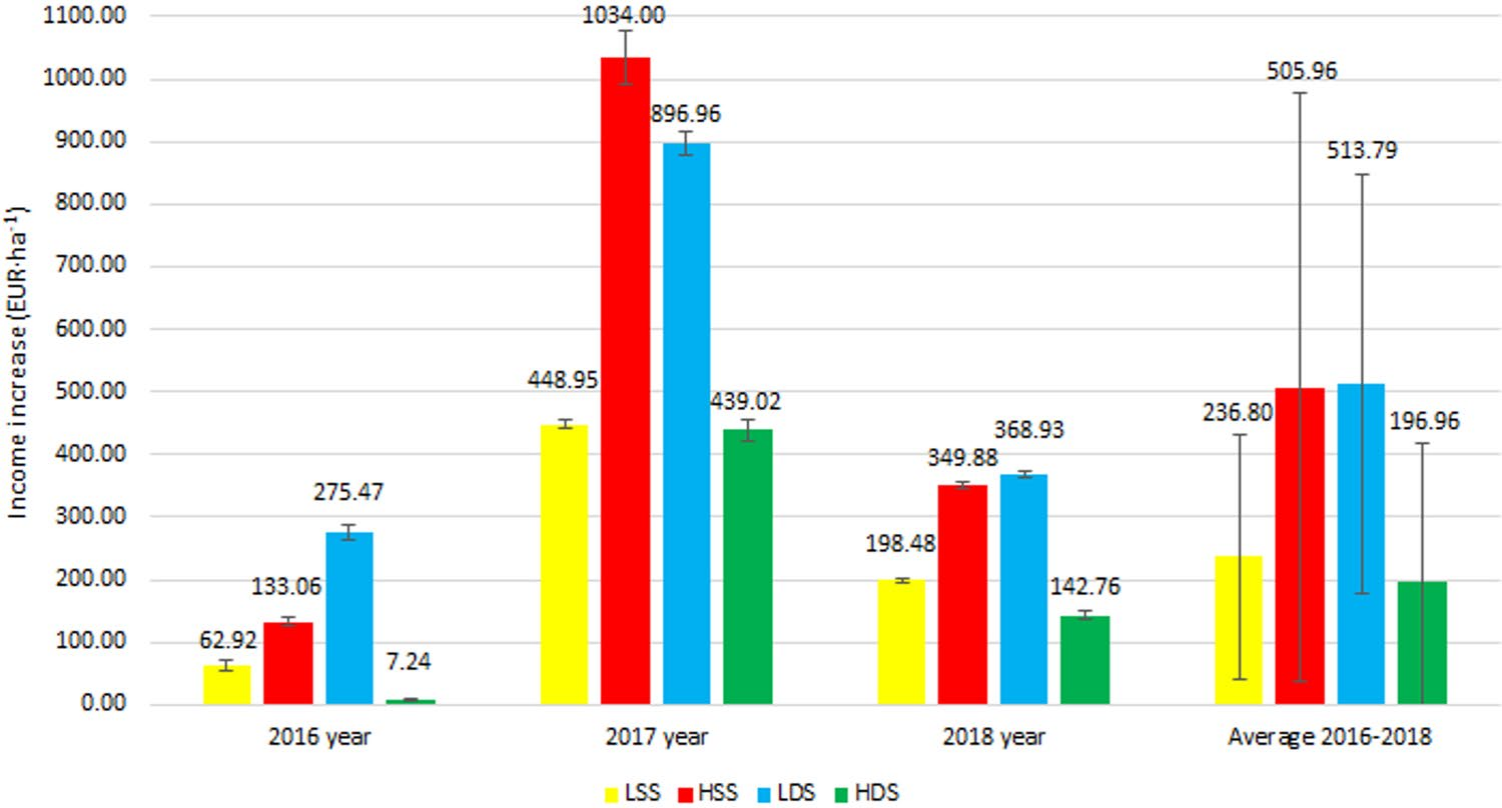
Economic effects of biostimulant Terra Sorb Complex use. *C* control, *LSS* lower concentration single spraying, *LDS* lower concentration double spraying, *HSS* higher concentration single spraying, *HDS* higher concentration double spraying.

The comparative analysis of the efficacy of various biostimulants and different methods of their application in common bean cultivation (Figs. 3, 4) is a type of tool aiding the choice of the cultivation system suitable for this plant. In all combinations tested, the application of two types of natural biostimulants had a positive impact on bean seed yield and, thus, on the increased cost-effectiveness of this crop cultivation.

In 2016, the profitability of biostimulants application ranged from 7.24 (for Terra Sorb Complex, Fig. 4) to 752.57 EUR·ha^−1^ (for Kelpak SL, Fig. 3). The highest profitability was demonstrated upon double foliar application of Kelpak SL in its higher concentration tested (HDS).

The use of Terra Sorb Complex in 2016 also brought some profits, they were however lesser and reached 119.67 EUR·ha^−1^ on average (Fig. 4). In the case of this biostimulant, a higher effectiveness was achieved after its double foliar application in the lower concentration (LDS). In turn, double plant spraying with Terra Sorb Complex in the higher analyzed concentration (HDS) ensured an income close to the limit of profitability (income at 7.24 EUR·ha^−1^) (Fig. 4).

Analyses conducted after the second year of bean cultivation regarding the effect of biostimulants and methods of their application on the economic profitability (Figs. 3, 4) demonstrated an increased income compared to the control samples (C). In the combinations of 2017, the total profitability from using these biostimulants ranged from 333.76 (for Kelpak SL, Fig. 3) to 1034.00 EUR·ha^−1^ (for Terra Sorb Complex, Fig. 4).

In the case of double plant treatment with Kelpak SL, the highest income was recorded upon its use in the higher concentration (HDS). The similar results were observed upon LSS treatment by this biostimulant.

In 2017, for Terra Sorb Complex, the greatest economic profits were noted compared to other years of field experiment. The total profitability from using this biostimulant was the highest in two cases of treatments—after HSS (1034.00 EUR·ha^−1^) and LDS (896.96 EUR·ha^−1^).

In the last year of the study, a higher income was noted from bean cultivation with the use of Kelpak SL biostimulant.

In 2018, the highest profitability was ensured by double plant spraying with the preparation containing seaweed in its lower concentration (LDS). Income reached the level of 819.07 EUR·ha^−1^ (Fig. 3). In the case of Kelpak SL application, the increase in income was also demonstrated after HDS and LSS treatment.

Different economic effects were observed after the application of Terra Sorb Complex in 2018. The average higher profitability was due to the LDS (368.93 EUR·ha^−1^) and HDS (349.88 EUR·ha^−1^) treatments. Despite the fact that the use of the Terra Sorb Complex biostimulant resulted in an increase in income, it was on average two times lower than in the case of the Kelpak SL biostimulant application.

Results of the present study show clearly that bean cultivation profitability was determined, above all, by the method of biostimulants application, and also by the composition of biostimulants and by the course of meteorological conditions in particular study years. The hypothesis assuming that the application of biostimulants is fully justified considering economic concerns was confirmed in all combinations of the biostimulants tested.

## Discussion

The results of the experiment demonstrate that the use of biostimulants, containing seaweed extracts and amino acids may ensure higher bean yield due to their multi-faceted action. In particular, presented results prove the significant effect of products application on the yield, as well as the nutritional and nutraceutical potential of seeds. In the case of Kelpak SL and Terra Sorb Complex, various responses of bean plants were noted. Therefore, comprehensive analysis of the obtained results, confirm biostimulants’ potency in modification of plant growth and development, expressed by increasing crop effectiveness^17,39^. According to Rouaphel et al.^40^, the higher productivity of the biostimulant-treated crops is ascribed, most of all, to the greater absorption of nutrients, osmotic regulation, and finally to increased contents of many secondary metabolites. However, diversified effects of biostimulant application indicated by agronomic parameters should also be reflected at the physiological and biochemical level. This was the case with our study, where both biostimulants induced major biochemical modifications, including in particular the antioxidative potential and concentrations of protein, starch and fiber in seeds.

Results of the present study demonstrate that the use of biostimulants evoked changes at the biochemical level, including enhanced synthesis of secondary metabolites. The changes observed in the antioxidative potential of bean seeds were usually greater upon the use of Kelpak SL biostimulant containing a seaweed extract. Shahabivand et al.^41^ even drew a conclusion that the higher total antioxidative capability of plants treated with biostimulants (especially in the form of spraying) helps them disperse photosynthetically produced electrons and alleviate oxidative damages. However, according to Fujita et al.^42^, changes in the biochemical profile of biostimulant treated plants can, primarily, be due to the increased concentration of abscisic acid (ABA) which is an indispensable phytohormone regulating various aspects of plant growth and development. Stirk et al.^12^ proved that the Kelpak SL contains ABA in a low concentration. There is also evidence that high cytokinin concentration can induce ABA biosynthesis, and thus the cytokinin content in synergy with the low ABA content in Kelpak SL may partially account for the observed positive response of bean plants, expressed by seed biochemical composition. Xiong et al.^43^ demonstrated that ABA content increased under stress conditions and induced the expression of many genes encoding various proteins important for not only the biochemical but also for the physiological processes. This has been confirmed in results obtained by Trivellini et al.^44^. A conclusion can, thus, be drawn from study results reported by the aforementioned authors that a decreased concentration of abscisic acid in plants treated with a biostimulant is most likely due to the inactivation of ABA signal pathways that control stomata closure.

In addition, the present study showed a significant decrease in proline concentration in seeds from plants treated with tested biostimulant. Proline occurs in various plants, including especially these exposed to stress conditions. It has been implied to serve multiple physiological functions including osmoregulation, energy and nitrogen absorption, but also to perform as an ageing signaler and the so-called stress sensor^41^. However, some researchers question the straight relationship between proline concentration and adaptation to stress^45^. Accumulation of this amino acid can also occur under physiological non-stress conditions (in situations of increased demand for protein synthesis)^46^. It is therefore possible that the variable levels of proline in bean seeds were not due to stress but to the application of biostimulants. The obtained data showed that in the most of the analyzed seeds combination the concentration of proteins increased with the decrease of proline content. On the other hand, some reports are available in scientific literature about a correlation between contents of proline and soluble phenols in plants. Cheynier et al.^47^ advanced a research hypothesis that proline synthesis may be accompanied by additional NADPH oxidation. As a consequence of the above, the increased NADP+NADPH ratio can determine the enhanced activity of the oxidative pathway of pentose phosphate, thereby assuring precursors for the phenolic biosynthesis via the shikimic acid pathway^41,48^. A study conducted by Rouphel et al.^49^ demonstrated proline concentration in biostimulant treated plants to contribute to better osmotic regulation. In addition, such antioxidative enzymes as CAT and GPX exhibit various activities in plants treated with biostimulants. The activity of GPX and—most of all—of CAT, which is responsible for degradation of intracellular hydrogen peroxide^50^, was significantly higher in plants treated with such preparations. Results obtained by Rouphael et al.^49^ suggest the use of biostimulants activates both proline and antioxidative enzymes, which represents some kind of plant strategy for counteracting oxidative damages under normal and stress conditions.

**Figure 5:**
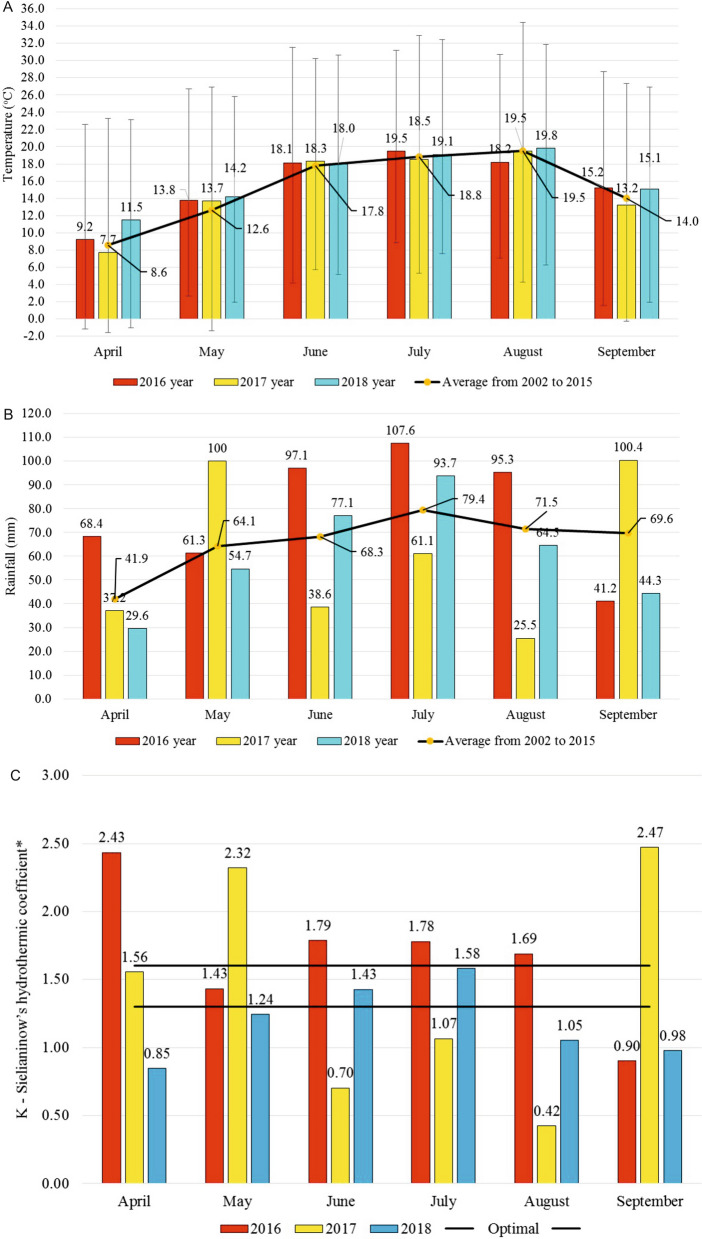
The average temperature, rainfalls and hydrothermal indicator Sielianinowa in the bean growing season. *Coefficient value: < 0.40 extremely drought; 0.40–0.70 very drought; 0.71–1.00 drought; 1.01–1.30 quite drought; 1.31–1.60 optimal; 1.61–2.00 relatively humid; 2.01–2.50 humid; 2.51–3.00 very humid; > 3.00 extremely humid.

Our study showed also a significant increase in protein concentration in bean seeds, especially upon the plant treatment with the biostimulant based on free amino acids. Providing the plants with an additional source of amino acids through biostimulants aids the plant defense mechanism which prevents water loss and plasmolysis. In our study, this was of particular importance in the second year of the field experiment. It is shown that plants were then exposed to stress, caused by too low levels of rainfall (Fig. 5). The Sielianinow’s hydrothermic coefficient even indicates that in the months critical for the development of beans, the plants were exposed to drought. The use of preparations based on free amino acids stimulates the photosynthesis process and, by this means, determines the rate and direction of metabolic processes^51^. An additional outcome of amino acids activity is the feasibility of internal hormonal and enzymatic regulation in plants. As claimed by Ertani et al.^52^, this is also determined by the enhanced activity of glutamine synthetase and glutamate synthase, which are active at a specified concentration of NH_4_^+^, which ultimately can increase nitrogen concentration in plants. Results of the research carried out by Bettoni et al.^53^ indicate biostimulant application to lead to the improved absorption and translocation of nitrogen from roots to shoots in plants, which explains the increased protein content of leaves and seeds^41^.

It should be emphasized that both the biostimulants based on seaweed extracts and these based on amino acids affect the development and resistance of particular parts of plants, i.e.: rhizosphere (roots), phyllosphere (green parts and shoots), and spermosphere (flowers and fruit), and by this means determine the quantity and quality of crop yield produced. Hence, the application of biostimulants ensures positive effects due to the complicated and multiple roles of their biologically active compounds. First of all, they stimulate many processes like e.g. root development, seed germination or chlorophyll production and photosynthesis, thereby increasing plant yield and plant resistance to stress conditions through, e.g. increasing the production of phenols^54^. The biostimulants were also proved to be able to induce the activity of PAL enzyme which is a key regulator of phenolic compounds biosynthesis^52^. Hypotheses advanced by Ertani et al.^55^ assume that PAL activity was also determined by an increased content of polyphenols in paprika, especially at the maturity stage. In addition, such an increase in the content of phenolic compounds is, probably, responsible for the higher FRAP values^55–57^.

Frequent abiotic stress leads to the excessive synthesis of reactive oxygen species (ROS), which can cause extensive cell damage. ROS are usually rapidly removed owing to antioxidative mechanisms, however this process may be suppressed by stress^58^, causing an increase in their intracellular concentration and greater damages. However, plant cells are able to prevent or repair such damages through a complex defense system including a series of protective genes associated with the antioxidative stress and leading to changes in the plant’s biochemical profile. Both, ROS production and antioxidative processes evoke synergistic, additive or antagonist effects on oxidative stress control^59^. According to Wozniak et al.^60,61^ biostimulants influence the production of ROS in plant cells. They have an effect under both stressful and optimal conditions. However, many authors emphasize that ‘a golden mean’ principle should be followed while using preparations in plant cultivation. Although results of multiple studies have shown the production of secondary metabolites in plants (like polyphenolic compounds) to be a beneficial phenomenon, it is so however only till the moment when the specific homeostasis is upset in plants. Therefore, the goal of biostimulant application in crop cultivation is, most of all, to aid many natural defense systems of plants to ensure detoxication or to prevent the adverse effects of ROS and potential stresses^62,63^. In our research the results concerning the accumulation of starch and crude fiber in seeds were surprising. It has been shown that both tested biostimulants increased the fiber content while reducing the total starch level. Los et al.^64^ indicates that this may be a positive effect of the biostimulants application, as the observed trend may have benefits for human health. This is due to the fact that high fiber content may reduce the speed and degree of digestibility of starch.

In addition, the positive response of bean plants to the application of biostimulants, can be attributed to the regulation of phytohormone activity, especially in the case of Kelpak SL^65^. It should be pinpointed that the induction of fiber synthesis in plants is controlled by plant hormones, including auxin, as well as gibberellin and cytokinin^66,67^. Increased contents of fiber in bean seeds may be due to the action of auxins and gibberellins from Kelpak SL, which control the formation and structure of lignins in the cell walls^68^. These mentioned phytohormones are considered specific signaling compounds, which induce the process of fiber synthesis and accumulation in various components of plant cells and tissues^69,70^.

It needs to be emphasized, however, that responses to stress are not just simple pathways but complex, combined systems that include multiple pathways^71^. Therefore, responses of plants triggered by the biostimulants applied differ between families, genera, and species, and thus it is essential to investigate genetic and molecular drivers of their effects. According to Gómez-Merino^71^, this approach will represent a research area of great importance to the future scientific investigations. Such an approach is definitely substantiated by both, food safety and sustainable development or by the effective use of the current inputs^72^.

The present study proved the natural biostimulants tested to positively modulate both the size and quality of crop yield. However, additional financial analysis was carried out to evaluate economic attractiveness of the biostimulating preparations that would enable their wider and rational use in the agricultural practice. Only when the application of biostimulants ensures not only higher yields but also higher incomes for farmers, will their potential be fully exploited. The economic analysis demonstrated that both preparations tested allowed increasing the profitability of bean cultivation, however a better effect was achieved on average with Kelpak SL used in the form of double spraying in the higher concentration (HDS) of the working solution.

Results obtained in this study provide a useful tip and, at the same time, a practical recommendation suitable in bean cultivation, especially in developing countries where the agricultural sector has a considerable contribution to the GDP. The understanding of not only mechanisms of action of the biostimulants but also of the profitability of their application may prove helpful in the development of policies supporting crop cultivation at minimized or eliminated use of chemical plant protection agents. Profitability assessment is often neglected in research works devoted to the application of plant growth regulators, while it is one of the most frequently accepted indicators of the economic activity in an enterprise. Therefore, it should be emphasized that profitability analyses usually provide highly important information about the outcomes of choosing and implementing a certain agricultural technology at a farm^73^.

According to Mariano^74^, such an approach to the evaluation of the impact of using growth regulators would encourage a greater number of farms to engage in the sustainable agriculture through the use of biostimulants. Investigations conducted so far have mainly been focused on the quantitative determination of growth promotion and crop yield increase after biostimulant application^75,76^. A complex approach to this problem was presented by Mariano^74^, who estimated expenditures and incomes of a farm determined by the use of a caragenin biostimulant in rice production. His study demonstrated that the application of this preparation improved the gross margin of the farmer. Implementation of this agronomic treatment modified the contribution of production cost elements. In this case, the costs of labor constituted the majority of expenditures; they were followed by costs incurred on the purchase and application of biostimulants, and finally by the expenditures associated with the purchase of sowing materials. As claimed by Mariano^74^, the use of fertilizers was minimized owing to the use of the biostimulant. In addition, the economic indicators determined in the study showed that the expected profits exceed the predicted costs. Similar observations were made by Jesus^77^, who evaluated the effect of using a biostimulant containing effective microorganisms on the economic profitability of maize cultivation and demonstrated that its application increased the profitability and margin of sugar maize cultivation.

The above considerations may be recapitulated with conclusions from the study conducted by Zhang and Schimidt^78^, who demonstrated that good physiological effects can be achieved by using small doses of biostimulants, which results in higher yield and quality of crops, and ultimately—in higher incomes for the farmers. In addition Crepaldi^79^ and Jesus et al.^77^ emphasized the fact that it is extremely important to provide the farmers with the information about costs of this type of cropping system in order to optimize the use of resources in a cultivation cycle and to achieve better productivity.

The conducted study demonstrates that the natural biostimulants tested constitute an effective tool to be used in bean cultivation management in order to stimulate plant growth and productivity. Their application under conditions of unpredictable climatic changes represents a sustainable and environment-friendly agronomic practice. Noteworthy is, however, the necessity of continuous development and extension of knowledge on their effects and on responses of specified crops to them. The study results demonstrate not only a significant increase in bean yield, but also modification of the chemical composition of seeds compared to the control samples. According to the obtained data, application of both biostimulants increased the yield of bean, but better results were observed after the use of Kelpak SL. In conclusion, the application of tested preparations significantly influenced on nutritional and nutraceutical quality of bean seeds. Terra Sorb Complex caused the highest increase in proteins level. In the light of achieved data, both biostimulants in similar level decreased the accumulation of starch. In opposite the most promising results in the context of nutraceutical value of bean, was obtained in the case of increasing level of fiber. Most importantly, a positive impact of both biostimulants on the seeds antioxidant potential was noted, expressed by the increased synthesis of phenolics, flavonoid, anthocyanins and antioxidant activities. A better average effect was observed upon the use of Kelpak SL biostimulant.

While considering the impact of the application method of biostimulants on the traits analyzed, double plant spraying with these preparations in their higher concentration (HDS) was found recommendable.

Study results demonstrate also significant differences in the economic profitability. In this case, the highest income earned by the farmers was achieved by double plant spraying with Kelpak SL biostimulant in its higher concentration (HDS).

Results of the conducted experiment can represent an element of support for the implementation of an agroecological tool in bean cultivation management, especially considering the possibility of reducing expenditures on chemical plant protection agents, savings, and providing farmers new opportunities for sustainable plant nutrition. Nevertheless, this approach would require vast changes in the rational agricultural practices and abandoning the faith in pesticides and fertilizers for the benefit of integrated methods of biotic and abiotic stress management in crop cultivation. This may be facilitated by results of this study, which directly indicate economic benefits from the use of biostimulants, which is extremely important to the farmers. In turn, from the consumers’ perspective it is highly important that the implementation of this agronomic practice offers them food products with an increased nutraceutical potential.

## Methods

### Plant materials and growth conditions

A field experiment was performed in the years 2016–2018 in Perespa village (50° 66′ N; 23° 63′ E, Poland) with common bean (*Phaseolus vulgaris* L.) of Mexican Black cultivar. The experiment was established in a random block system, in 4 replications, on experimental plots with the surface area of 10 m^2^. Bean was cultivated on the soil belonging to the Gleyic Phaeozems, which was characterized by an alkaline pH (pH in 1 M KCl: 7.4–7.5). Contents of available nutrients in the soil were at medium levels: P (12.6–14.2 mg P_2_O_5_ in 100 g of soil), K (15.3–17.1 mg K_2_O in 100 g of soil), Mg (6.2–6.8 mg Mg in 100 g of soil), and N (8.1–9.3 mg N–NO_3_ + N–NH_4_ in 100 g of soil). Bean seeds were sown on the 2nd May of 2016, 2017, and 2018, with 3.5 cm gaps in rows with 45 cm spacing. No herbicides were used, and weeds were removed mechanically and manually. The fertilization and irrigation were not carried out. In particular growing seasons, bean plants were sprayed with Kelpak SL and Terra Sorb Complex biostimulants, according to the scheme presented in Table 3. Plants sprayed with water (being a solvent to the biostimulants used) served as the control.

**Table 3:** Plant developmental stages and dates of biostimulant application.

Biostimulant	Biostimulant composition^14,80^	Number of sprays and plant developmental stages (BBCH) in which the biostimulants were applied	Concentration (%)	Volume of working solution/working pressure
Kelpak SL	Auxins (11 mg L^−1^), cytokinins (0.031 mg L^−1^), alginates (1.5 g L^−1^), amino acids (total 441.3 mg 100 g^−1^), mannitol (2261 mg L^−1^), neutral sugars (1.08 g L^−1^). Macroelements (N 0.09%, P 90.7 mg kg^−1^, K 7163.3 mg kg^−1^, Ca 190.4 mg kg^−1^, Mg 337.2 mg kg^−1^, Na 1623.7 mg kg^−1^). Microelements Mn 17.3 mg kg^−1^, Fe 40.7 mg kg^−1^, Cu 13.5 mg kg^−1^, Zn 17.0 mg kg^−1^, B 33.0 mg kg^−1^)	Single spraying: BBCH 13–15 (LSS)	0.7	300 L ha^−1^/0.30 MPa
		Single spraying: BBCH 13–15 (HSS)	1.0	
		Double spraying: BBCH 13–15, BBCH 61 (LDS)	0.7	
		Double spraying: BBCH 13–15, BBCH 61 (HDS)	1.0	
Terra Sorb Complex	Aliphatic amino acids (glycine, alanine, valine, leucine, isoleucine, proline). Hydroxyamino acids (serine, threonine). S containing amino acids (cysteine, methionine). Aromatic amino acids (phenylalanine, tryptophan, tyrosine). Acidic amino acids (aspartic acid, glutamic acid). Basic amino acids (histidine, arginine, lysine). Organic N (5.0%), B (1.5%), Mg (0.8%), Fe (1%), Zn (0.1%), Mn (0.1%), Mo (0.001%), and many microelements	Single spraying: BBCH 13–15 (LSS)	0.3	300 L ha^−1^/0.30 MPa
		Single spraying: BBCH 13–15 (HSS)	0.5	
		Double spraying: BBCH 13–15, BBCH 61 (LDS)	0.3	
		Double spraying: BBCH 13–15, BBCH 61 (HDS)	0.5	

*BBCH* Biologische Bundesanstalt, Bundessortenamt and Chemical industry; *BBCH 13–15* 3 leaves unfolded. *BBCH 61* beginning of flowering: approximately 10% of flowers open, *LSS* lower concentration single spraying, *HSS* higher concentration single spraying, *LDS* lower concentration double spraying, *HDS* higher concentration double spraying.

Biostimulants were used in terms when the foliar application of microelements is recommended. Their doses were established based on the authors’ experience from previous investigations^35,38^.

In the BBCH 89 stage (bean full maturity: ripe pods, hard seeds with typical coat color to the cultivar), plants were harvested from plots. In 2016 and 2018, the harvest took place on August 10, and in 2017—August 5.

The average temperature, rainfalls and hydrothermal indicator Sielianinowa in the bean growing season are shown in Fig. 5.

### Phenolics content and antioxidant capacity determination

Ground been seeds were subjected to the extraction process with a mixture of acetone, water, and hydrochloric acid (70:29:1, v/v/v)^81^. The samples were centrifuged for 10 min (6800 × *g*). The process of extraction was conducted in three replications for each analyzed combination of seeds. After centrifugation, supernatants were collected, combined, and used for further laboratory analyses.

### Determination of total phenolic compounds (TPC)

The total pool of phenolic compounds (TPC) was determined using a Folin–Ciocalteau reagent. The seed extract was mixed with water (0.5:0.5, v/v) and then 2 mL of the Folin–Ciocalteau reagent (1:5 H_2_O), and afterwards 10% sodium carbonate were added to the mixture. After 30 min, absorbance of the samples was measured at a wavelength of 724 nm using a UV–Vis spectrophotometer. The total concentration of phenolic compounds was expressed in mg of gallic acid equivalents (GAE) per g of dry matter (DM)^82^.

### Determination of flavonoid content (TFC)

The total pool of flavonoids (TFC) was determined acc. to the method described by Lamaison and Carnet^83^. The seed extract was mixed with a 2% methanolic solution of AlCl_3_ × 6H_2_O (1:1, v/v). After 10-min incubation at room temperature, absorbance of the solutions was measured at a wavelength of 430 nm using a UV–Vis spectrophotometer. The concentration of flavonoids was expressed in mg of quercetin equivalents (QE) per g of dry matter (DM).

### Determination of anthocyanins (TAC)

The content of anthocyanins in bean seeds was determined acc. to the method provided by Fuleki and Francis^84^. Determinations were carried out using solutions of potassium chloride and sodium acetate at two pH values, i.e. 1.0 and 4.5. The solutions were mixed with bean seed extracts in a ratio of 20:1 (v/v). After 15 min, absorbance of the samples was measured at two wavelengths (520 nm and 700 nm). After absorbance value correction for various pH values, the content of anthocyanins was expressed in mg of cyanidin 3-glucoside equivalents (Cy3-GE) per g of dry matter (DM).

### Reducing power

Bean seed extracts were mixed with a 200 mM phosphate buffer (pH 6.6) and a 1% aqueous solution of K_3_ [Fe (CN_6_)], in a ratio of 1:1:1 (v/v/v). Then, the mixtures were incubated at a temperature of 50 °C. After 20 min, 0.5 mL of trichloroacetic acid was added to the mixture. Next, the samples were centrifuged (6800 × *g*, 10 min), and the collected supernatants were combined and mixed with distilled water and a 0.1% aqueous solution of iron (III) chloride (2.5:2.5:0.5, v/v/v). Absorbance of the samples was measured at a wavelength of 700 nm using a UV–Vis spectrophotometer. The reducing power was expressed in mg of Trolox equivalents per g of dry matter (DM)^85^.

### FRAP

The experiment was conducted following the method proposed by Jimenez-Alvarez et al.^86^ with some modifications. The FRAP mixture was obtained by mixing 300 mM acetate buffer (3.6 pH), TPTZ (10 mM dissolved in 40 mM HCl), and FeCl_3_ × 3H_2_O (10:1:1, v/v/v). Next, 25 µL of the sample, extraction mixture (blank sample), Trolox (calibration solution), and 250 µL of the FRAP mixture were transferred to a 96-well microplate. The whole mixture was pipetted and incubated at room temperature for 8 min. Sample absorbance was measured at a wavelength of 593 nm, and the result obtained was expressed as Trolox equivalent in mL of the solution.

### ABTS

The antiradical activity of bean seed extracts was determined acc. to the method provided by Sancho et al.^87^ with some modifications. A Trolox calibration solution (0, 25, 50, 75, 100, 150, 200, 300 µM/mL) was prepared in the extraction mixture. An ABTS·+ solution with the final concentration of 2.45 mM potassium persulfate and 7 mM ABTS·+ was diluted with an acetone solution to ensure the absorbance of 0.7 ± 0.02 at 734 nm. Then, 280 µL of ABTS·+ were transferred to a 96-well microplate and mixed with 20 µL of the sample. The result obtained was expressed as Trolox equivalent in mL of the solution.

### Proline

Proline content was determined acc. to the method proposed by Carillo and Gibon^88^ using a 1% ninhydrin solution. In brief, 50 µL of the bean seed extract were added to 100 µL of the reaction mixture, and the sample was incubated at a temperature of 95 °C for 20 min. Then, the samples were cooled to a room temperature and centrifuged at 2500 rpm for 1 min. Next, 100 µL of the solution were transferred to a 96-well microplate and absorbance was measured at a wavelength of 520. The content of proline was expressed in µM per mL.

### Protein

Protein content was determined based on the method proposed by Redmile-Gordon et al.^89^ with own modifications. The Bradford reagent was transferred to a 96-well microplate, and mixed with the analyzed samples, standard protein (BSA) or the reaction mixture (blank sample). The final volume of the samples was 200 µL. Afterwards, the samples were incubated on a shaker at a room temperature for 15 min. The value of extinction measured at 595 nm was read out using an Epoch Microplate Spectrophotometer (BioTek—USA).

### Total starch

The content of total starch was evaluated according to the method presented by Goñi et al.^90^ 1997. 50 mg samples were dispersed in 2 M KOH (6 mL to each sample). Samples were shaken at temperature 20 °C (30 min). After this process the hydrolisis of the starch was conducted by adding sodium acetate buffer (pH = 4.75) and amyloglucosidase (1 mg mL^−1^; 14 U mg^−1^). The samples were incubated for 45 min at 60 °C in water bath with shaking. The starch was calculated as glucose (mg) × 0.9.

### Crude fiber

The crude fiber analysis was performed according to the AOCS Approved Procedure Ba 6a-05^91^, using filter bag technique (Ankom 200). The fiber was determinate as the organic residue remaining after digesting with sulfuric acid (0.255 N) and sodium hydroxide (0.313 N) and ashing for 2 h at 600 °C. Filter bags F57 (filter media chemically inert and heat resistant) were used (Ankom Technology).

### Economic analysis

The economic effect of biostimulants application was computed based on the value of yield increase resulting from the use of biostimulants and costs of their application^92^.

Income growth resulting from the use of biostimulants (*O*_*sb*_) was calculated from the following formula:

1 $$ O_{sb} = W_{pp} - K_{sb} , \left( {{\text{EUR}}\,{\text{ha}}^{ - 1} } \right), $$

where *W*_*pp*_ is the value of yield increase, (EUR ha^−1^), *K*_*sb*_ is the costs of biostimulants use, (EUR ha^−1^),

The value of yield increase (*W*_*pp*_) was computed acc. to the following formula:

2 $$ W_{pp} = (P_{nb} - P_{nk} ) \cdot C_{n} ,{ }\left( {{\text{EUR}}\,{\text{ha}}^{ - 1} } \right), $$

where *P*_*nb*_ is the seed yield from the combination with biostimulant application, (t ha^−1^), *P*_*nk*_ is the seed yield from the control combination, (t ha^−1^), *C*_*n*_ is the average price of seeds in a given study year, (EUR t^−1^).

Costs of the use of biostimulants (*K*_*sb*_) were computed acc. to the following formula:

3 $$ K_{sb} = k_{b} + k_{w} + k_{z} ,{ }\left( {{\text{EUR}}\,{\text{ha}}^{ - 1} } \right), $$

where *k*_*b*_ is the cost of biostimulant purchase, (EUR ha^−1^), *k*_*w*_ is the cost of water used for the treatment, (EUR ha^−1^), *k*_*z*_ is the cost of performing the treatment, (EUR ha^−1^).

The average purchase price was established based on information from wholesale markets (934.58 EUR t^−1^). The cost of purchasing biostimulants was calculated as a mean price from 3 wholesale companies supplying farms (Kelpak SL 10.98 EUR L^−1^; Terra Sorb Complex 18.69 EUR L^−1^). Water use cost was calculated based on the price of 1 m^3^ of tap water in a village community in the Lubelskie Province (1.87 EUR m^−3^). Cost of treatment performance was calculated as a mean price of the spraying service with a 1000 L lift-mounted sprayer (14.02 EUR ha^−1^).

### Statistical analysis

Analyses were performed in three replications for each growing season. The Shapiro–Wilk test was used to evaluate the normal distribution of data. Results were analyzed using the one-way analysis of variance (ANOVA). The significance of differences between mean values was estimated based on Tukey confidence intervals, at a significance level of p < 0.05. For the reported data the standard deviation value (SD) was determined. The statistical analysis was performed using Statistica 13.3 software (TIBCO Software Inc., USA)^93^.
